# Early Detection of Mild Cognitive Impairment With In-Home Sensors to Monitor Behavior Patterns in Community-Dwelling Senior Citizens in Singapore: Cross-Sectional Feasibility Study

**DOI:** 10.2196/16854

**Published:** 2020-05-05

**Authors:** Iris Rawtaer, Rathi Mahendran, Ee Heok Kua, Hwee Pink Tan, Hwee Xian Tan, Tih-Shih Lee, Tze Pin Ng

**Affiliations:** 1 Department of Psychiatry Sengkang General Hospital Singhealth Duke NUS Academic Medical Centre Singapore Singapore; 2 Department of Psychological Medicine Yong Loo Lin School of Medicine National University of Singapore Singapore Singapore; 3 School of Information Systems Singapore Management University Singapore Singapore; 4 Duke-NUS Graduate Medical School Singapore Singapore

**Keywords:** dementia, neurocognitive disorder, pattern recognition, automated/methods, internet of things, early diagnosis

## Abstract

**Background:**

Dementia is a global epidemic and incurs substantial burden on the affected families and the health care system. A window of opportunity for intervention is the predementia stage known as mild cognitive impairment (MCI). Individuals often present to services late in the course of their disease and more needs to be done for early detection; sensor technology is a potential method for detection.

**Objective:**

The aim of this cross-sectional study was to establish the feasibility and acceptability of utilizing sensors in the homes of senior citizens to detect changes in behaviors unobtrusively.

**Methods:**

We recruited 59 community-dwelling seniors (aged >65 years who live alone) with and without MCI and observed them over the course of 2 months. The frequency of forgetfulness was monitored by tagging personal items and tracking missed doses of medication. Activities such as step count, time spent away from home, television use, sleep duration, and quality were tracked with passive infrared motion sensors, smart plugs, bed sensors, and a wearable activity band. Measures of cognition, depression, sleep, and social connectedness were also administered.

**Results:**

Of the 49 participants who completed the study, 28 had MCI and 21 had healthy cognition (HC). Frequencies of various sensor-derived behavior metrics were computed and compared between MCI and HC groups. MCI participants were less active than their HC counterparts and had more sleep interruptions per night. MCI participants had forgotten their medications more times per month compared with HC participants. The sensor system was acceptable to over 80% (40/49) of study participants, with many requesting for permanent installation of the system.

**Conclusions:**

We demonstrated that it was both feasible and acceptable to set up these sensors in the community and unobtrusively collect data. Further studies evaluating such digital biomarkers in the homes in the community are needed to improve the ecological validity of sensor technology. We need to refine the system to yield more clinically impactful information.

## Introduction

### Background

Dementia is a neurodegenerative disease of epidemic proportions and incurs substantial burden on the affected families and the health care system. Globally, about 47 million people were living with dementia in 2015, and this number is projected to triple in 2050. The global costs of dementia in 2015 were estimated at US $818 billion, a 35.4% increase compared with 2010. Up to 1 in 5 of the community-dwelling older adults aged 65 years and above suffer from mild cognitive impairment (MCI) [1]. MCI is considered as an at-risk state for dementia, a stage when the affected person is likely responsive to appropriate interventions. Existing evidence suggests that multidomain, multicomponent interventions can improve or maintain cognitive function of persons with MCI, delaying further cognitive decline [2]. If dementia onset is delayed by 5 years, then it would halve the global dementia prevalence and would result in substantial reduction in medical, family, and social care burden for dementia. There is an urgent need for the early detection of MCI to facilitate monitoring and intervention and to allow individuals and their families to plan ahead. According to the practice guidelines for MCI by the American Academy of Neurology [3], detecting MCI early, performing serial assessments and implementing interventions, and allowing individuals to plan ahead are essential.

Delayed recognition of MCI in the community is a missed opportunity for early intervention. One challenge that clinicians face is the reliance on patients or their relatives for reporting subtle changes or decline, and these often occur when the cognitive decline is relatively advanced. As a result, only a fraction of individuals with MCI seek early medical attention. There are many undiagnosed MCI cases in the community who would only seek help when they have more fulminant symptoms of dementia, thus missing the opportunity of having early interventions to delay the progression to dementia. In rapidly ageing societies, working adult children cannot be relied upon to detect these subtle changes; hence, innovative methods have to be employed to be our *eyes and ears* in the community.

Technology can be these *eyes and ears*. Indeed, sensor technology is quickly gaining popularity in the medical community for its utility in continuous health monitoring [4]. In contrast to the extensive literature on the usage of sensors to detect falls, assess gait, and remotely monitor physical health [5-7], the use of sensors to monitor cognition and mental well-being is comparatively less well studied. Extant studies are often laboratory based and conducted in test beds or facilities [4,8,9]. A systematic review of home-based monitoring of cognitive function published this year indicated that few studies have done real-life evaluations in uncontrolled conditions [10]. Some earlier studies looked at continuous recording of daily audio patterns and linked it to social and mental well-being [11], whereas others looked at utilizing mobile phone sensors to evaluate depressive symptom severity and physiological signals associated with mental, emotional, and physical stresses [12,13]. Specific to cognition, one group approached early detection of dementia by using infrared sensors to monitor in-house activities; they found that subjects with impaired cognition had lesser number of outings and a shorter sleep time compared with controls [14]. This same group replicated their findings in a bigger sample over the course of 1 year and found that senior citizens who had shown cognitive decline had lesser outings [15]. Similar passive in-home sensor setups were reported in other two studies evaluating cognitive states [16,17]. The coefficient of variation of median walking speeds in the MCI group was twice that of controls [16], and there were distinct trajectories of walking speeds between different cognitive states over 2.6 (SD 1.0) years of follow-up [17].

To establish feasibility and acceptability of remote monitoring of senior citizens’ behavior patterns in our community, we conducted a pilot study utilizing multiple sensors installed at home to capture certain behaviors. These behaviors would typically be assessed in a clinical evaluation, such as forgetfulness, sleep, and activity levels. We hypothesized that (1) sensor-derived data of specific activity patterns between 2 groups of community-dwelling seniors, those with MCI and those who are cognitively healthy (healthy cognition; HC), will differ and (2) sensors for remote monitoring in the homes of senior citizens would be acceptable.

## Methods

### Study Design and Participant Recruitment

This was a cross-sectional study conducted over a period of 2 months. The study commenced in March 2016 and was completed in August 2018. Participants were recruited from existing community studies such as the Singapore Longitudinal Ageing Study and Jurong Ageing Study (JAS). These participants had previously consented to be recontacted for related studies. Participants were also recruited from the community through senior citizen activity centers such as Presbyterian Community Services. Institutional ethics review board approvals were obtained (reference number: 2015/01076).

Informed consent was obtained before participants were screened for eligibility. Participants were included if they were (1) aged between 65 and 85 years, (2) living alone, (3) able to provide written informed consent in English/Mandarin, and (4) available for the entire duration of the study. Participants were excluded if they (1) had a previous diagnosis of dementia/any neurodegenerative condition, (2) had a diagnosis of any psychiatric disorder, (3) had limitations of physical mobility or required assistance with their activities of daily living, or (4) were not willing to have sensors deployed in stipulated areas of the home.

### Data Collection

At baseline, basic sociodemographic data were collected. Depressive symptoms were elicited using the Zung Self-Rating Depression Scale (SDS) and Geriatric Depression Scale (GDS). Subjective sleep quality was captured using the Pittsburgh Sleep Quality Index (PSQI). The Friendship Scale (FS) was used to capture social connectedness. Measures of global cognition including the Montreal Cognitive Assessment (MoCA) and modified Mini-Mental State Examination (MMSE) were administered. Participants who had not been administered the Clinical Dementia Rating (CDR) or neuropsychological test batteries within the past 6 months (as part of other studies) performed these tests. Structured Clinical Interview for DSM Disorders was administered to all participants. Participants who screened positive for any DSM disorder were excluded. SDS, GDS, PSQI, FS, MMSE, and MoCA were repeated at 2 months. Participants were asked for feedback at the end of the 2 months of participation. This was an unstructured qualitative written feedback.

Participants’ cognitive status of HC vs MCI was established at baseline with the MMSE, MoCA, CDR, and neuropsychological test performance and via a consensus panel.

The *diagnosis of MCI* was defined according to the following published criteria: (1) subjective memory and cognitive difficulties or informant/clinician-observed cognitive difficulties; (2) objective cognitive impairment in one or more domains: MMSE global score ranging from 24 to 27 and at least one neurocognitive domain (attention, memory, executive function, language, or visuospatial abilities) with a score of 1 to 2 standard deviations less than the age- and education-adjusted mean values; (3) CDR scale global score >0.5; (4) essentially independent in performing basic activities of daily living; and (5) not demented. Subtyping into amnestic and nonamnestic MCI based on the presence or absence of memory impairment was done according to established criteria [18].

### Sensor Setup and Behaviors Captured

**Figure 1:**
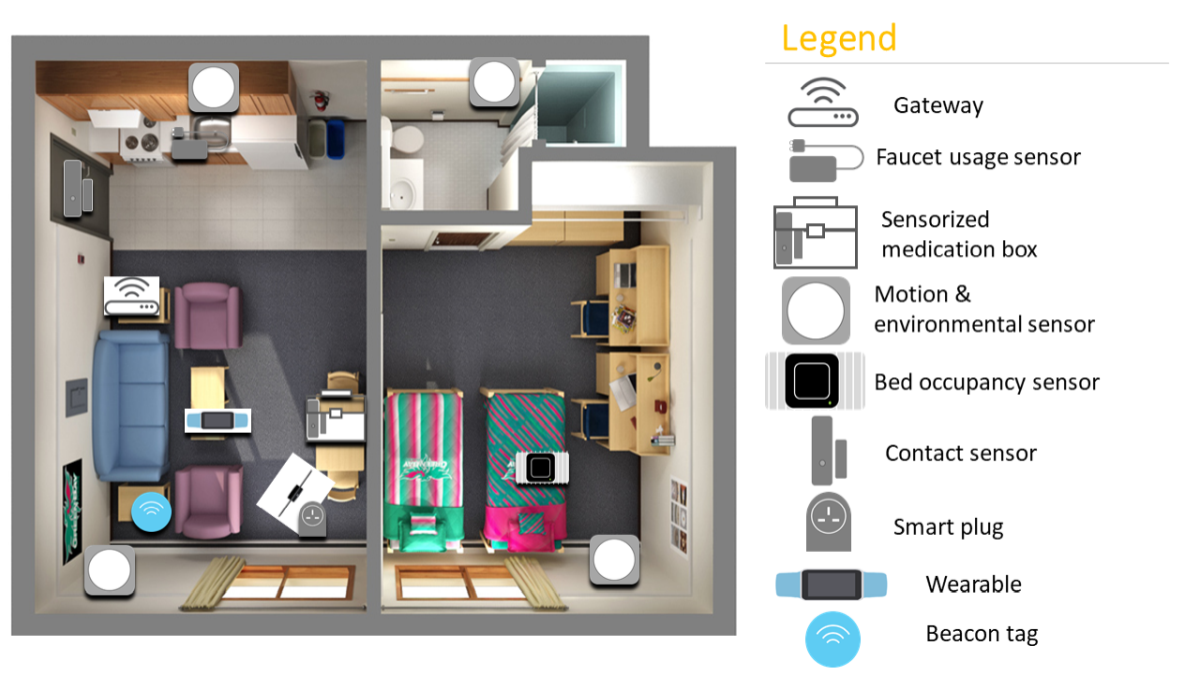
Multimodal sensor set up in homes.

**Figure 2:**
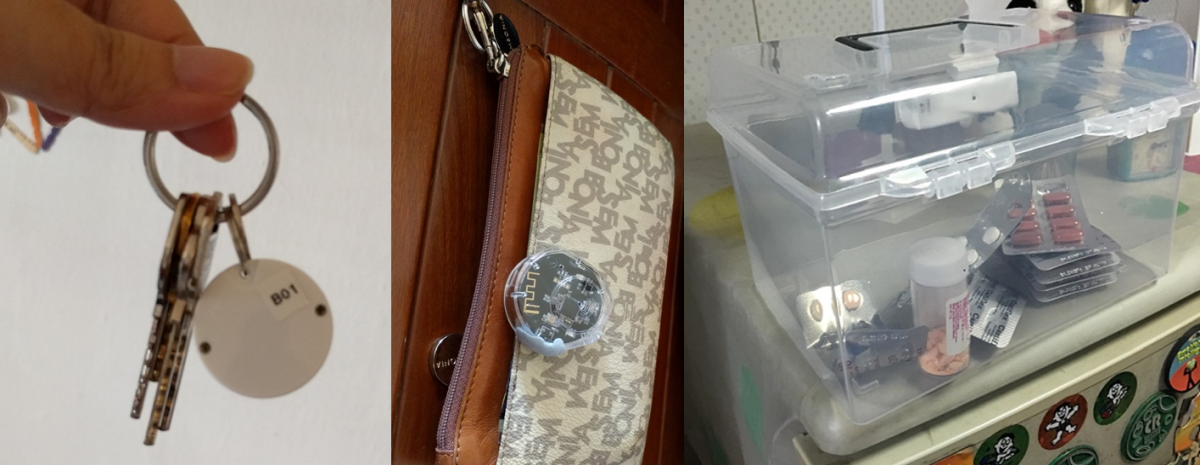
Proximity beacon tags to wallet and keys and sensor-equipped medication box.

Upon completion of baseline assessments, participants had their homes instrumented with a network of sensors (Figure 1) for a duration of 2 months. The multimodal sensor system comprised passive infrared (PIR) motion sensors, proximity beacon tags, a sensor-equipped medication box (forgetfulness; Figure 2), a bed sensor (sleep), and a wearable (pedometer and heart rate). Each sensor periodically sensed the physical environment and then wirelessly transmitted the sensed data to the gateway. The gateway transmitted the aggregated data to the backend server via secure cellular communications (eg, 3G) for monitoring and processing. Each data point was identifiable only via the sensor node identifier; the mapping between the sensor node identifier and the home was securely stored and accessible only by the study investigators.

The sensor network was used to capture several behaviors of interest. The main feature/outcome of interest was *forgetfulness*. The other features were in-home activity levels, sleep quality, and physical activity; other changes are seen in cognitive decline but are often overlooked.

A combination of sensor data was used to measure forgetfulness. Participants were provided with a sensor-equipped medication box to store all their prescription medication; data were generated whenever the box was opened [19]. These data, taken together with the expected medication frequency information obtained at baseline, allowed us to determine the number of times a participant forgot to take their medication at the prescribed time. Proximity beacon tags were attached to participants’ personal effects such as keychains and wallet, allowing us to estimate the distance between the item and the home gateway. Efforts were made at baseline to ensure that these personal effects were items that were routinely brought out. Coupled with the wearable and PIR motion sensors, we were able to determine if the participant had forgotten to bring these items with them when leaving home. The faucet usage sensor was used to determine if the participant had forgotten to switch off the faucet after moving away from the designated area (detected by motion sensors). The in-home activity levels and number of outings were inferred from the PIR motion sensors and door contact sensor, which detects opening and closing of the main door of the participant’s residence. The bed sensor (based on fiberoptic technology) placed under the participant’s mattress provided data on sleep duration and quality. The wearable activity band (Microsoft band) measured heart rate and daily steps. Participants were expected to wear the band at all times, with the exception of shower time. The smart plug was used to detect if specific appliances in the home were used, most commonly the television [20].

### Safety

Although detection of no movement was not an aim of this study, given that it is a capability of the system, we provided this feature for safety monitoring as all the senior citizens enrolled were living alone. As such, if the system detected no movement for 8 hours, an alert was sent to the caregiver, care provider (social service agency), or research team.

### Sample Size Justification

An earlier study with a sample size of 14 comparing walking speeds and variance in MCI and healthy controls using continuously obtained data from sensors revealed a significant difference [16]. Given the feasibility and resource constraints of the study, we planned a priori to enroll 36 participants at a minimum.

### Data Analysis

Raw sensor data readings were converted into a common format and aggregated into a database. Purging of the data was performed to remove erroneous data and periods where the system was down/partially down. It was necessary to remove days where the system was down, as it would affect the *forgetfulness* metric. After data purging, sensor-specific data cleaning or validation was performed to ensure that only valid sensor data are processed. Frequency of each metric was computed, for example, frequency of forgetting medication per month, frequency of outings per day, and frequency of sleep interruptions per night. Investigators involved in obtaining sensor data and analysis were blinded to participants’ cognitive status.

Descriptive statistics for demographic information and psychometric scores were computed for all participants at baseline. As psychometric measures were administered at baseline and at 2 months, these scores were averaged to provide a precise cross-sectional estimate. Demographic, psychometric characteristics, and behavior metrics (computed from sensor data) were compared between the HC participants and MCI participants using the Student *t* test or the Wilcoxon rank sum test for continuous variables and the Pearson chi-square test and the Fisher exact test for categorical variables. Depending on the distribution of data, Pearson or Spearman tests were applied to look at a correlation between sensor-derived data and psychometric test measures.

## Results

### Participant Characteristics

A total of 59 participants were screened. One participant was ineligible because of an existing mental health condition. Another 8 participants were not enrolled because of reasons of hospitalization, family objections, or overseas travel during the study period. One participant was enrolled but dropped out shortly after the sensors were deployed because of discomfort with the bed sensor. In total, 49 participants completed the study. Of the 49 participants, 28 were diagnosed with MCI and 21 with HC. Half of the MCI participants were of the amnestic subtype and half were of the nonamnestic subtype. Participant demographics and psychometric measures are shown by group in Table 1. There were no statistically significant differences in demographics between the completers (n=49) and the noncompleters (n=10).

**Table 1:** Participant characteristics by group—normal cognition vs mild cognitive impairment.

Demographics		Cognitively healthy (n=21)	Mild cognitive impairment (n=28)
Age (years), mean (SD)		73.0 (5.3)	75.1 (6.3)
**Gender, n (%)**			
	Male	7 (33)	9 (32)
	Female	14 (67)	19 (68)
Years of education, mean (SD)		7.0 (4.0)	4.5 (3.9)
Employment (currently employed part time), n (%)		8 (38)	2 (7)
Housing type—Housing Development Board 1-2 room flat, n (%)		14 (67)	20 (71)
**Marital status, n (%)**			
	Never married	8 (38)	10 (36)
	Separated/divorced	7 (33)	9 (32)
	Widowed	5 (24)	9 (32)
**Medical conditions, n (%)**			
	Hypertension	11 (52)	17 (60)
	Hyperlipidemia	12 (57)	19 (68)
	Diabetes mellitus	4 (19)	6 (22)
	Stroke	0 (0)	3 (11)
	Ischemic heart disease	2 (10)	4 (14)
**Psychometric measures, mean (SD)**			
	Mini-Mental State Examination	28.1 (3.2)	26.3 (2.2)
	Montreal Cognitive Assessment	27.5 (1.6)	24.0 (3.1)
	Geriatric Depression Scale	0.6 (0.7)	1.4 (1.0)
	Zung Depression Scale	44.5 (2.1)	42.7 (3.4)
	Pittsburg Sleep Quality Index	3.8 (3.2)	5.0 (2.2)
	Friendship Scale	18.5 (1.7)	19.1 (1.7)

### Behaviors of Interest and Psychometric Measures

Frequencies of incidents of forgetfulness and various behaviors of interest (as described above) were computed and compared between the MCI and HC groups (Table 2).

As expected, the MCI group had lower MMSE scores than the HC group. They also tended to have poorer sleep quality, with higher scores on the PSQI. With the sensor-derived data, we found that MCI participants were less active than their HC counterparts; MCI participants had an average of 3407 steps a day compared with 4033 steps in the HC group. MCI participants spent less time away from home daily. They had more sleep interruptions per night (2 per night) compared with the HC group (1 per night). The MCI group had forgotten their medications an average of two times more per month compared with the HC group (30 vs 28). The MCI group forgot their wallet when leaving the home at a similar frequency to the HC group. Unexpectedly, the HC group had a higher frequency of forgetting their keys per month as compared with the MCI group. It is important to note that none of these differences achieved statistical significance. Faucet use data were not analyzed as the sample size of usable data was too small; there were many implementation issues with the sensor. Correlation analysis of sensor-derived behavior metrics with psychometric measures did not yield any significant results.

**Table 2:** Comparisons of activities between the cognitively healthy group and mild cognitive impairment group.

Behaviors of interest, mean (SD)	Cognitively healthy (n=21)	Mild cognitive impairment (n=28)	*P* value
Steps (daily)	4033 (2148)	3407 (2688)	.40
Heart rate (bpm)	72 (4)	75 (7)	.22
Sleep duration daily (min)	440 (155)	427 (246)	.84
Number of sleep interruptions	1 (1)	2 (2)	.27
Number of outings daily	1 (1)	1 (1)	.93
Time away from home daily (min)	300 (153)	267 (132)	.44
Frequency of forgetting medication/month	28 (13)	30 (28)	.85
Frequency of forgetting keys per month	21 (16)	17 (13)	.40
Frequency of forgetting wallet per month	24 (17)	24 (22)	.94
Television use daily (min)	174 (176)	219 (220)	.52

### Acceptability

A total of 83% (41/49) of the participants gave positive feedback at the study conclusion. Many participants found it reassuring that the system was able to detect deviations in their daily activity patterns and liked it that someone was aware if they declined physically or psychologically. Many asked if there was an option for the system to be permanently installed. Some negative feedback included needing to charge the wearable, worrying about electricity costs, and finding the water usage sensor inconvenient. Selected positive and negative feedback obtained from the participants at study conclusion are shown in Textbox 1.

**Selected positive and negative written feedback from the participants about the system.**

- “The sensor system is very good for me who is weak and old and living alone. In case anything happens to me, I know someone will help me, feel very secure and safe. The staff who came to install the sensor are very friendly, kind and helpful. The watch does not cause any inconvenience to me all. Instead, I feel very safe to go out alone knowing that my movements are being monitored. It will be better if I have the sensors installed at my place permanently.”
- “(1) With the sensors, feel secure - can sleep better. (2) Not used to the blinking light. (3) Found trouble in wearing the sensor watch. (4) Feel uncomfortable with the gadget near bathroom.”
- “The sensor system is very good for senior citizens living alone, feel very secure knowing my movements are being monitored all the time. The watch is very good. I can go out anytime. It will record my movements. One of my old neighbours was staying upstairs alone. The lady died in her toilet but her body was discovered many days later. If only she had the sensor system installed then she will be able to get help earlier.”
- “System is unobtrusive. Sensors are small and do not inconvenience me in any way. Enjoy the use of the Microsoft band. Does not alter her activities because of sensors. Bed sensor does not disturb her and she thinks she sleeps as per her sleep patterns. Thinks it is a useful system for elderly who live alone.”
- “In home sensors is very good for senior citizens living alone. In case anything happens to me, I am able to get help. Feel secure with sensors installed. The watch is good, can monitor my steps. Feel safe to go out alone. Disadvantage: - Electricity bill increased a little. Too many wires that take up space.”
- “In-home sensor is very helpful for senior citizens living alone. Multiple sensors installed taking up some of the space in the house. The one near the kitchen sink interfere with my daily washing, unable to clean properly, otherwise no other problem.”
- “I feel very safe and relax with sensors on. If possible, I would like to have the sensors installed in my house permanently. The whole system does not cause any problems to me at all.”
- “On a whole, quite satisfied with the sensors installed except I don’t like one of the sensors installed near my kitchen sink, one of the tubing extending halfway into the washing bowl (sink) causing inconvenience when washing my kitchen utensils. Hope this project able to help those elderly living alone in future.”
- “(1) Very satisfied with the sensors installed. (2) Like the watch as it can monitor my steps. (3) Does not cause any inconvenience. (4) Feel more secure and comfortable. (5) Overall, I feel good and happy and even request for extension. (6) Hope to have sensors installed permanently.”
- “I found sensors are very good. Every day when I came back, sensor lights will flicker, and I will feel very safe.”
- “Basically, it’s good to be monitored for safety reasons. I don’t find it a nuisance, but electricity is left on for 2 months.”
- “(1) Sensor is OK. (2) A bit of inconvenience with the sensor at the sink. (3) I seldom wear the watch when I go out.”
- “Good, not interfering with daily living but use more electricity.”
- “not suitable for me; suitable for blur and not very smart people; troublesome to wear the watch all the time; recommend for those very forgetful people”
- “Satisfied with the gadgets. Helpful for monitoring.”
- “Scared of the gadget with all the plugs and lighting.”
- “I feel very safe and relax with the sensors installed in my home; I also feel safe to go out wearing the watch; the sensor gadget does not interfere with my daily activities; good for elderly living alone.”
- “sensor system is good; monitor any movements at home to detect anything unusual; the watch is also good to monitor number of steps and movements outside my home and I can go out as usual with no restriction; no problems with all the sensor gadgets installed at home; highly recommended for elderly living alone.”

## Discussion

### Principal Findings

The results of this pilot study indicate that it is feasible to set up sensor networks in the homes of community-dwelling seniors and unobtrusively collect potentially meaningful clinical data. Differences between HC and MCI in several behaviors of interest including daily activity (step count and time away from home), sleep (duration and interruptions), and forgetting medication suggest that cross-sectional remote observation of behaviors can yield discernible patterns, albeit not achieving statistical significance. Our observations on activity and sleep measures are consistent with earlier studies [14,15] and existing understanding of MCI [21,22]. Some collected sensor-derived data were counter-intuitive—minimal differences in frequency of forgetting wallet and higher frequency of forgetting keys in HC. A possible explanation for these observations may be that the study period of 2 months was short; hence, lower-frequency events are less likely to demonstrate identifiable patterns. Half of the MCI participants were of the nonamnestic subtype; hence, they may not have demonstrated differences in the forgetfulness behavior metric. With a longer observation period, behavioral markers that are better indicators of cognitive impairment can be determined.

Given our initial concerns that senior citizens would be wary of remote monitoring systems and past research on unobtrusive systems indicating privacy or security concerns [23], over 80% of the participants giving positive feedback and finding the system acceptable was an immensely encouraging result. Negative feedback was related to practical user issues with specific devices such as having to charge the wearable, blinking lights of the motion sensor, and the need for multiple plugs. Despite the largely positive reception of the system from participants, we are careful not to extrapolate this acceptability to all senior citizens in general. The senior citizens in this study were all living alone and over two-thirds lived in 1-2 room public housing, a proxy of lower socioeconomic status. They are a group most at risk of undetected cognitive decline and most likely to benefit from an unobtrusive home monitoring system. Many existing sensor systems on trial by social care agencies for this group are specifically set up to detect falls or death, outcomes that sometimes go unnoticed in this group of vulnerable senior citizens.

### Strengths and Limitations

Strengths of this pilot study include the use of an entirely unobtrusive system without the use of cameras, protecting the privacy of participants. Moreover, clinically useful behavior metrics such as forgetfulness, activity levels, and sleep were captured. These sensors were trialed in actual residential homes and not in laboratory settings or assisted living facilities; senior citizens were observed in their natural environments with no change to their lifestyle. Unobtrusive observation in the senior citizens’ own homes highlights the potential translational value of having technology be the *eyes and ears* to monitor the senior citizens’ health without taxing the working adult population.

This study was planned to evaluate feasibility and was exploratory in nature. It is limited by its small sample size and short duration of observation. There was some data loss in the initial phases of the study when the system was down. This was quickly addressed, and the system uptime was at 80% to 90% for much of the study duration. The accuracy of some of the behavior metrics that act as proxies for *forgetfulness* will need to be refined in future studies. Missed medication doses may indicate both the intentional nonadherence to prescribed doses of medication or genuine forgetfulness. In future studies, apart from capturing baseline medication intake frequency, there should be a measure of baseline adherence to various medications. Similarly, the behavior metric of forgetting personal effects will also require fine-tuning in subsequent studies. Forgetting to switch off the faucet is a commonly asked clinical question. Unfortunately, the technical trial of the water usage sensor was not successful in this study. Finally, participants were recruited from previous cohort studies as well as senior citizen activity centers, and this may have led to selection bias. Moreover, the inability of motion sensors to differentiate between 2 unique individuals led to an inherent selection bias, with only individuals living alone being recruited.

### Conclusions

We found that it was both feasible and acceptable to use sensors to unobtrusively monitor behavior patterns in the homes of community-dwelling senior citizens. A larger study with a longer observation period of 2 to 3 years is underway. Negative feedback from the pilot has been addressed as far as practicable, including using fewer sensors and a different wearable. Analysis of trajectories and variability over time will yield more useful information, including behavior patterns that predict decline from MCI to dementia. Artificial intelligence methods including supervised learning models will be applied. Moving forward, we need to look at reducing the number of sensors to obtain more information. To improve generalizability to all senior citizens rather than only those who live alone, innovative solutions are needed to circumvent limitations of the motion sensor while still maintaining privacy. Identifying sensor-derived behavior metrics that do away with the motion sensor will allow the inclusion of senior citizens who live with others, improving the scalability of this solution. Although the system does not comprise video cameras, addressing privacy and security concerns is paramount when implementing and refining remote monitoring systems. Utilizing Internet of Things and artificial intelligence to monitor cognitive and physical well-being should be further developed to deliver value-added health care for senior citizens. Early detection of anomalies allows for self-management, timely interventions in the home and community, and facilitating remote capture of clinically meaningful data that can be utilized by health care professionals for diagnostic and prognostic purposes.

## Abbreviations

CDR: Clinical Dementia Rating
FS: Friendship Scale
GDS: Geriatric Depression Scale
HC: healthy cognition
JAS: Jurong Ageing Study
MCI: mild cognitive impairment
MMSE: Mini-Mental State Examination
MoCA: Montreal Cognitive Assessment
PIR: passive infrared
PSQI: Pittsburgh Sleep Quality Index
SDS: Self-Rating Depression Scale
